# Adaptive laboratory evolution of cadmium tolerance in *Synechocystis* sp. PCC 6803

**DOI:** 10.1186/s13068-018-1205-x

**Published:** 2018-07-24

**Authors:** Chunxiao Xu, Tao Sun, Shubin Li, Lei Chen, Weiwen Zhang

**Affiliations:** 1 Laboratory of Synthetic Microbiology, School of Chemical Engineering & Technology, Tianjin University, Tianjin, 300072 People’s Republic of China; 2 Key Laboratory of Systems Bioengineering, Ministry of Education of China, Tianjin, 300072 People’s Republic of China; 3 Collaborative Innovation Center of Chemical Science and Engineering, Tianjin, People’s Republic of China; 4 Center for Biosafety Research and Strategy, Tianjin University, Tianjin, People’s Republic of China

**Keywords:** Cadmium, Cyanobacteria, Adaptive laboratory evolution, Genome re-sequencing, Cross tolerance

## Abstract

**Background:**

Cadmium has been a significant threat to environment and human health due to its high toxicity and wide application in fossil-fuel burning and battery industry. Cyanobacteria are one of the most dominant prokaryotes, and the previous studies suggested that they could be valuable in removing Cd^2+^ from waste water. However, currently, the tolerance to cadmium is very low in cyanobacteria. To further engineer cyanobacteria for the environmental application, it is thus necessary to determine the mechanism that they respond to high concentration of cadmium.

**Results:**

In this study, a robust strain of *Synechocystis* PCC 6803 (named ALE-9.0) tolerant to CdSO_4_ with a concentration up to 9.0 µM was successfully isolated via adaptive laboratory evolution over 802-day continuous passages under cadmium stress. Whole-genome re-sequencing was then performed and nine mutations were identified for the evolved strain compared to the wild-type strain. Among these mutations, a large fragment deletion in *slr0454* encoding a cation or drug efflux system protein was found to contribute directly to the resistance to Cd^2+^ stress. In addition, five other mutations were also demonstrated related to the improved Cd^2+^ tolerance in ALE-9.0. Moreover, the evolved ALE-9.0 strain was found to obtain cross tolerance to some other heavy metals like zinc and cobalt as well as higher resistance to high light.

**Conclusions:**

The work here identified six genes and their mutations related to Cd^2+^ tolerance in *Synechocystis* PCC 6803, and demonstrated the feasibility of adaptive laboratory evolution in tolerance modifications. This work also provided valuable information regarding the cadmium tolerance mechanism in *Synechocystis* PCC 6803, and useful insights for cyanobacterial robustness and tolerance engineering.

**Electronic supplementary material:**

The online version of this article (10.1186/s13068-018-1205-x) contains supplementary material, which is available to authorized users.

## Background

In recent years, environmental pollution caused by heavy metals has caused serious problems, including contaminating water, entering food chain and posing threats to growth of living organisms in nature [1, 2]. Among them, cadmium ion (Cd^2+^) is one of the most dangerous heavy metals [3]. Cadmium residues mainly come from industrial products like nickel–cadmium battery and pigmenting, which inevitably pollute a large amount of water [4]. Nowadays, cyanobacteria, which have been considered as “photosynthetic microbial factories” in the biosynthesis of fine chemicals and biofuels, have attracted much attention [5]. On the other hand, as one of the most dominant prokaryotes on Earth, cyanobacteria play a pivotal role in the global carbon cycling [6], while were threatened sometimes by the unfriendly environment. For example, it was reported that the cadmium concentration could reach 0.36 ± 0.82 mg/L (3.2 ± 7.3 µM) in the industrial area of Penang, Malaysia [7], which would pose significant threat to the survival of cyanobacteria. Thus, it is essential to understand how they respond to environmental stresses such as Cd^2+^. In addition, removal of toxic metal ions such as Cd^2+^ from water by cyanobacteria has been widely evaluated in recent years and is considered as a promising alternative treatment in wastewater purification [8]. For instance, a study on adsorption of Cd^2+^ by *Gloeothece magna* suggested that they would probably be cultivated in water bodies contaminated by Cd^2+^ to ameliorate its toxicity effectively [9]. Therefore, it will be of great value to decipher the tolerance mechanism to Cd^2+^ in cyanobacteria.

Cellular responses of cyanobacteria to high concentration of Cd^2+^ have been investigated in the past decades. In a previous study, the direct influence of Cd^2+^ to photosynthetic machinery was found to be multiphase effects in model cyanobacteria *Synechocystis* sp. PCC 6803 (here after *Synechocystis*). The results showed that Cd^2+^ first limited photosystem I acceptor side, and 7 h later, it disturbed photosystem II under the existence of light [10]. In addition, Cd^2+^ toxicity caused the generation of reactive oxidative species (ROS) and the consumption of glutathione as well as the thiol-group containing protein [11]. Furthermore, Cd^2+^ penetrated rapidly into the cells and replaced other heavy metals like Ca^2+^ and Zn^2+^ by competing for enzymes and disturbing membrane potential [12]. In another study, Cd^2+^ triggered the integrated reprogramming of the whole metabolism in *Synechocystis*, which was controlled by the Slr1738 regulator [13]. Meanwhile, some genes were found involved in resistance to Cd^2+^, like *smtA* in *Synechococcus* PCC 7942 [14] and *sll0649* in *Synechocystis* [8]. Together, cellular responses to Cd^2+^ toxicity involved a variety of complex reaction mechanisms. However, up to now, the detailed mechanism of Cd^2+^ toxicity to cyanobacteria has still not been fully understood. Although some algae like *Phormidium ambiguum* and *Scenedesmus quadricauda var. quadrispina* show good tolerance to Cd^2+^ at the concentration of up to 0.35 mM [15], they are non-model organisms and relatively difficult to be deeply studied, while *Synechocystis* sp. PCC 6803 is a model organism with known genomic information [16] and relatively easily genetic operation, although it showed poor tolerance to Cd^2+^ stress [8]. Therefore, it is necessary to enhance the Cd^2+^ tolerance of *Synechocystis* and understand how the Cd^2+^ tolerance is regulated, so that to guide the tolerance engineering in other algal or cyanobacterial species.

Adaptive laboratory evolution (ALE) is a strategy to improve strains via constant batch transfer under specific growth conditions [17], and has been considered as a powerful tool to generate robust strains with enhanced tolerance to multiple stresses [18]. Although it is time-consuming for strains to accommodate, systematic modifications on genome scale could be obtained through the ALE process [19]. Predictably, ALE could also bring other consequences like trade-off in growth or cross tolerance in alternative environments [18]. In addition, mature high-throughput sequencing and genetic manipulation systems make ALE possible for mechanism research and even further phenotypes modification [20]. In *Saccharomyces cerevisiae*, four strategies to isolate cobalt-tolerant cells were performed by ALE, yielding the most resistant mutant to cobalt stress from 2.5 to 8 mM, which indicated the efficiency of ALE to improve strains [21]. In addition, ALE was employed successfully in *Synechocystis* to improve 1-butanol tolerance from a concentration of 0.2–0.5% (v/v) and a further metabolomic basis for rational tolerance engineering was determined [22].

In this study, the ALE strategy was employed to improve the tolerance of *Synechocystis* to Cd^2+^ stress. As a result, after 128 continuous passages of approximately 802 days, tolerance of the evolved strain was improved from 4.6- to 9.0-µM Cd^2+^. With the aid of high-throughput re-sequencing technology, the mutations in the genome of the evolved strain compared with the wild-type strain were identified and further functionally characterized. This study demonstrated the feasibility of ALE in tolerance modifications and provided useful insights for cyanobacterial robustness and tolerance engineering.

## Results

### Adaptive laboratory evolution of Cd^2+^ tolerance in *Synechocystis*

**Fig. 1:**
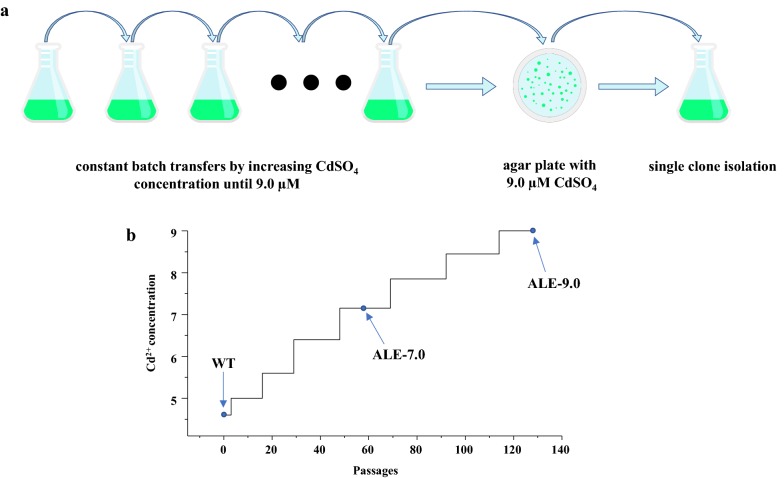
Experimental setup of ALE process in this study. **a** Increasing CdSO_4_ concentration was from 4.6 to 9.0 µM. Agar plate supplemented with 9.0-µM CdSO_4_ was then used to isolate single clone. **b** Simplified evolution process of Cd^2+^ tolerance in *Synechocystis*. The *x* axis represented passages and *y* axis represented the simplified Cd^2+^ concentration. The specific evolved concentrations of cadmium were 4.6, 5.0, 5.4, 5.8, 6.2, 6.6, 7.0, 7.3, 7.7, 8.0, 8.3, 8.6, and 9.0 µM. Three points represented three strains for sequencing in this study. *ALE* adaptive laboratory evolution

Wild-type (WT) *Synechocystis* strain was evolved by serial passaging for 128 passages (802 days) in BG11 medium supplemented with CdSO_4_, as a selective pressure to enrich population with Cd^2+^ tolerance. The starting Cd^2+^ concentration for WT was set as 4.6 µM as our previous study showed that WT showed a slight growth deficiency at this Cd^2+^ concentration level [23]. Under normal BG11 medium without CdSO_4_, *Synechocystis* could achieve late exponential phase (OD_750 nm_ = 1.5) from an initial inoculum (OD_750 nm_ = 0.1) within 96 h. When CdSO_4_ was added, cell growth rate decreased obviously. In this ALE process, a simple rule was established that once the evolutionary strain could reach OD_750 nm_ of 0.5 (from an initial inoculum of OD_750 nm_ of 0.1) within 96 h, the Cd^2+^ concentration was increased. Thus, the *Synechocystis* strain was cultivated with increasing Cd^2+^ concentration from 4.6 µM gradually to 9.0 µM (Fig. 1a). Figure 1b demonstrates the simplified process of the ALE experiment, as a gradually increasing concentrations of Cd^2+^ process. Finally, after 128 continuous passages of 802 days, a strain that could tolerate 9.0-µM Cd^2+^ was obtained, approximately 95% improvement in tolerance compared with WT. At the end of ALE process, the evolved strain was plated on BG11 agar plate supplemented with 9.0-µM CdSO_4_. Four single colonies were cultured individually under 9.0-µM CdSO_4_ and the one which showed the fastest growth (data not shown; named ALE-9.0) (Table 1) was selected for further study.

**Table 1:** Strains and plasmids used in this study

Name	Description	Restriction site(s)	References
EZ-T™	A cloning vector transformed from pBlueScript II SK(+)	NA	GenStar
pCP3031	A suicide vector integrated between *slr2030* and *slr2031*	NA	[24]
*E. coli* DH5α	F^−^, φ80d *lac*Z∆M15, ∆ (*lac*ZYA-*arg*F) U169, *deo*R, *rec*A1, *end*A1, *hsd*R17(rk^−^, mk^+^), *pho*A, *sup*E44, λ-, *thi*-1, *gyr*A96, *rel*A1	NA	TransGen Biotech
WT	Wild-type *Synechocystis* sp. PCC 6803	NA	ATCC 27184
ALE-7.0	A evolved strain just tolerant to 7.0-µM CdSO_4_	NA	This study
ALE-9.0	End-point strain of ALE	NA	This study
*∆slr0454* ^WT^	WT-*∆slr0454*::*cm*^r^	NA	This study
*∆slr0623* ^WT^	WT-*∆slr0623*::*cm*^r^	NA	This study
*∆slr0721* ^WT^	WT-*∆slr0721*::*cm*^r^	NA	This study
*∆slr0774* ^WT^	WT-*∆slr0774*::*cm*^r^	NA	This study
*∆slr0798* ^WT^	WT-*∆slr0798*::*cm*^r^	NA	This study
*∆slr1302* ^WT^	WT-*∆slr1302*::*cm*^r^	NA	This study
*∆ssr1480* ^WT^	WT-*∆ssr1480*::*cm*^r^	NA	This study
*∆sll1586* ^WT^	WT-*∆sll1586*::*cm*^r^	NA	This study
*∆slr1753* ^WT^	WT-*∆slr1753*::*cm*^r^	NA	This study
WT-C	WT-∆slr0168::*cm*^r^	NA	This study
*∆slr0454* ^ALE-9.0^	ALE-9.0-*∆slr0454*::*cm*^r^	NA	This study
*∆slr0623* ^ALE-9.0^	ALE-9.0-*∆slr0623*::*cm*^r^	NA	This study
*∆slr0721* ^ALE-9.0^	ALE-9.0-*∆slr0721*::*cm*^r^	NA	This study
*∆slr0774* ^ALE-9.0^	ALE-9.0-*∆slr0774*::*cm*^r^	NA	This study
*∆slr0798* ^ALE-9.0^	ALE-9.0-*∆slr0798*::*cm*^r^	NA	This study
*∆slr1302* ^ALE-9.0^	ALE-9.0-*∆slr1302*::*cm*^r^	NA	This study
*∆ssr1480* ^ALE-9.0^	ALE-9.0-*∆ssr1480*::*cm*^r^	NA	This study
*∆sll1586* ^ALE-9.0^	ALE-9.0-*∆sll1586*::*cm*^r^	NA	This study
*∆slr1753* ^ALE-9.0^	ALE-9.0-*∆slr1753*::*cm*^r^	NA	This study
ALE-9.0-C	ALE-9.0-∆slr0168::*cm*^r^	NA	This study
OE-*slr0454*^WT^	*∆slr2030*–*slr2031*:: *P*_*cpc560*_*_ slr0454*^WT^ *_T*_*rbcl*_ *spe*^*r*^	*Xho*I/*Nde*I	This study
OE-*slr0623*^WT^	*∆slr2030*–*slr2031*:: *P*_*cpc560*_*_ slr0623*^WT^ *_T*_*rbcl*_ *spe*^*r*^	*Xho*I/*Bgl*II	This study
OE-*slr0721*^WT^	*∆slr2030*–*slr2031*:: *P*_*cpc560*_*_ slr0721*^WT^ *_T*_*rbcl*_ *spe*^*r*^	*Xho*I/*Bgl*II	This study
OE-*slr0774*^WT^	*∆slr2030*–*slr2031*:: *P*_*cpc560*_*_ slr0774*^WT^ *_T*_*rbcl*_ *spe*^*r*^	*Xho*I/*Bgl*II	This study
OE-*slr0798*^WT^	*∆slr2030*–*slr2031*:: *P*_*cpc560*_*_ slr0798*^WT^ *_T*_*rbcl*_ *spe*^*r*^	*Xho*I/*Bgl*II	This study
OE-*slr1302*^WT^	*∆slr2030*–*slr2031*:: *P*_*cpc560*_*_ slr1302*^WT^ *_T*_*rbcl*_ *spe*^*r*^	*Xho*I/*Bgl*II	This study
OE-*ssr1480*^WT^	*∆slr2030*–*slr2031*:: *P*_*cpc560*_*_ ssr1480*^WT^ *_T*_*rbcl*_ *spe*^*r*^	*Xho*I/*Bgl*II	This study
OE-*sll1586*^WT^	*∆slr2030*–*slr2031*:: *P*_*cpc560*_*_ sll1586*^WT^ *_T*_*rbcl*_ *spe*^*r*^	*Xho*I/*Bgl*II	This study
OE-*slr1753*^WT^	*∆slr2030*–*slr2031*:: *P*_*cpc560*_*_ slr1753*^WT^ *_T*_*rbcl*_ *spe*^*r*^	*Xho*I/*Bgl*II	This study
OE-*slr0454*^ALE-9.0^	*∆slr2030*–*slr2031*:: *P*_*cpc560*_*_ slr0454*^ALE-9.0^ *_T*_*rbcl*_ *spe*^*r*^	*Xho*I/*Bgl*II	This study
OE-*slr0623*^ALE-9.0^	*∆slr2030*–*slr2031*:: *P*_*cpc560*_*_ slr0623*^ALE-9.0^ *_T*_*rbcl*_ *spe*^*r*^	*Xho*I/*Bgl*II	This study
OE-*slr0721*^ALE-9.0^	*∆slr2030*–*slr2031*:: *P*_*cpc560*_*_ slr0721*^ALE-9.0^ *_T*_*rbcl*_ *spe*^*r*^	*Xho*I/*Bgl*II	This study
OE-*slr0774*^ALE-9.0^	*∆slr2030*–*slr2031*:: *P*_*cpc560*_*_ slr0774*^ALE-9.0^ *_T*_*rbcl*_ *spe*^*r*^	*Xho*I/*Bgl*II	This study
OE-*slr0798*^ALE-9.0^	*∆slr2030*–*slr2031*:: *P*_*cpc560*_*_ slr0798*^ALE-9.0^ *_T*_*rbcl*_ *spe*^*r*^	*Xho*I/*Bgl*II	This study
OE-*slr1302*^ALE-9.0^	*∆slr2030*–*slr2031*:: *P*_*cpc560*_*_ slr1302*^ALE-9.0^ *_T*_*rbcl*_ *spe*^*r*^	*Xho*I/*Bgl*II	This study
OE-*ssr1480*^ALE-9.0^	*∆slr2030*–*slr2031*:: *P*_*cpc560*_*_ ssr1480*^ALE-9.0^ *_T*_*rbcl*_ *spe*^*r*^	*Xho*I/*Bgl*II	This study
OE-*sll1586*^ALE-9.0^	*∆slr2030*–*slr2031*:: *P*_*cpc560*_*_ sll1586*^ALE-9.0^ *_T*_*rbcl*_ *spe*^*r*^	*Xho*I/*Bgl*II	This study
OE-*slr1753*^ALE-9.0^	*∆slr2030*–*slr2031*:: *P*_*cpc560*_*_ slr1753*^ALE-9.0^ *_T*_*rbcl*_ *spe*^*r*^	*Xho*I/*Bgl*II	This study
OE-C	*∆slr2030*–*slr2031*:: *P*_*cpc560*_*_T*_*rbcl*_ *spe*^*r*^	NA	This study

**Fig. 2:**
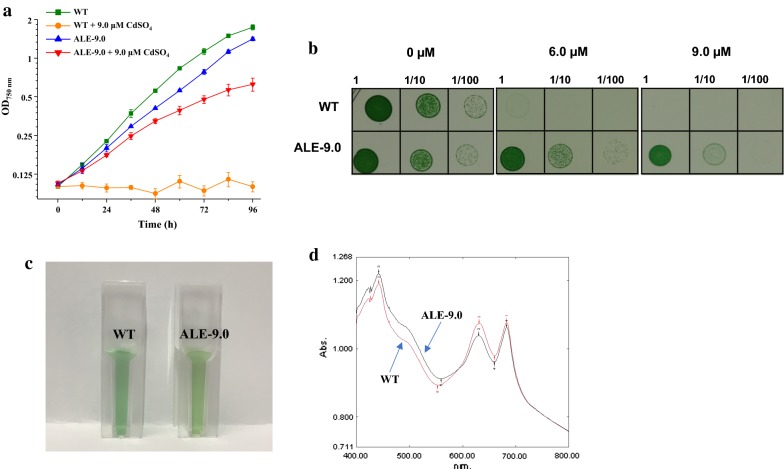
Comparisons between WT and ALE-9.0. **a** Growth patterns of WT and ALE-9.0 in normal BG11 medium or under 9.0-µM CdSO_4_. The error bars represented the calculated standard deviation of the measurements of three biological replicates. **b** Effect of different concentration of Cd^2+^ on WT and ALE-9.0 on BG11 agar plate. The upper line of each picture represented WT and lower one was ALE-9.0. **c** Color of WT and ALE-9.0 at OD_750 nm_ of 0.5 in normal BG11 medium. **d** Full absorption spectrum WT and ALE-9.0 in normal BG11 medium. Red curve represented WT and black curve represented ALE-9.0

As shown in Fig. 2a, under normal BG11 medium condition without Cd^2+^, ALE-9.0 grew slightly, but not significantly, slower than WT, while under 9.0-µM CdSO_4_ condition, the growth of ALE-9.0 was dramatically better than WT, as WT can hardly survive under 9.0-µM Cd^2+^, demonstrating the improved Cd^2+^ tolerance in ALE-9.0. To show the effect of ALE process better, tenfold serial dilutions of WT and ALE-9.0 liquid cultures were spotted onto BG11 agar plates with different concentrations of CdSO_4_ (Fig. 2b). Under normal BG11 medium, WT showed little difference in growth condition with ALE-9.0. However, once Cd^2+^ was added, WT could hardly survive under 6.0-µM Cd^2+^, while ALE-9.0 still remained robust growth even under 9.0-µM Cd^2+^. In addition, ALE-9.0 showed a little yellow–green phenotype under the normal illumination of 50-μ mol photons m^−2^ s^−1^. Full absorption spectrum indicated that ALE-9.0 had less phycocyanin at 625 nm [24] but more carotenoid around 505 nm [25] than WT in normal BG11 medium (Fig. 2c, d).

### Whole-genome re-sequencing of ALE-9.0

Notably, WT could only endure 4.6-µM CdSO_4,_ while ALE-9.0 could survive in medium supplemented with 9.0-µM CdSO_4_, indicating that some intrinsic genetic changes occurred during the ALE process. Whole-genome re-sequencing technology was then employed to identify the genomic differences between ALE-9.0 and WT. Genomes of ALE-9.0 and WT were re-sequenced and compared with reference from the database to check out the differences (https://www.ncbi.nlm.nih.gov/nuccore/NC_000911). In all, one deletion, nine single-nucleotide polymorphisms (SNPs) and four structural variations (SVs) were detected in ALE-9.0 compared to WT after the evolution process, and then, all these mutations were individually verified by Sanger sequencing. As a result, seven SNPs and one SV were identified, and the deletion turned out to be one insertion (Table 2). Among the confirmed nine mutations (seven SNPs, one SV, and one insertion), the insertion in *slr1753* was located in a high repetition region with low mapping quality, the SNP in *sll1586* was synonymous, while the others were non-synonymous. The only SV was a 659-bp-deletion located in the ORF of gene *slr0454*, which resulted in an early termination of a truncated *slr0454* encoding a protein of 574 amino acids (the original gene *slr0454* encoding a protein of 890 amino acids). It is speculative that these mutations might lead to an increased tolerance of *Synechocystis* to Cd^2+^ in the strain ALE-9.0.

**Table 2:** Mutations of the ALE-9.0 compared to WT

Position	Gene	Mutation		Protein	Mutated in 7.0 µM
		Nucleotide	Protein		
3506559	*slr0454*	SV	Early termination	Cation or drug efflux system protein	N
2961208	*slr0623*	T–C	P30L	Thioredoxin	Y
99738	*slr0721*	G–A	M113V	Malic enzyme	N
2401781	*slr0774*	A–G	G270R	Protein-export membrane protein SecD	Y
3061976	*slr0798*	G–A	Y207C	Zinc-transporting P-type ATPase involved in zinc tolerance	Y
306570	*slr1302*	G–C	P299A	Protein involved in constitutive low affinity CO_2_ uptake	N
1135407	*ssr1480*	G–A	Y24C	Putative RNA-binding protein	Y
1500784	*sll1586*	C–T	L496L	Unknown protein	Y
527831	*slr1753*	Ins(GAACCC)	1163PE	Hypothetical protein	Y

To investigate the roles of these mutations, one strain (ALE-7.0) evolved in the middle term of this evolution process and tolerant to 7.0-µM Cd^2+^ (Fig. 1b) was also selected, cultivated, and sequenced by Sanger sequencing concerning these mutations. The results showed that six out of these nine mutations found in ALE-9.0 were present in ALE-7.0 when CdSO_4_ concentration reached 7.0 µM. Thus, during the increasing concentrations of CdSO_4_ from 7.0 to 9.0 µM in this ALE, only three mutations (non-synonymous SNPs in *slr0721*, *slr1302* and the SV in *slr0454*) occurred in the later stage of the whole ALE process (Table 2), indicating their roles in later improvement of Cd^2+^ tolerance.

### Quantitative reverse transcription PCR (qRT-PCR) analysis of the mutated genes in WT and ALE-9.0

Besides the genetic differences between WT and ALE-9.0, the expression level of the mutated genes could possibly also change under CdSO_4_ stress conditions. To evaluate this hypothesis, five samples (WT cultured in normal BG11 medium and under 4.6-µM CdSO_4_, ALE-9.0 cultured in normal BG11 medium, under 4.6-µM CdSO_4_ and 9.0-µM CdSO_4_) were selected for qRT-PCR analysis. The expression change was presented by the ratio of the relative expression level of the genes under stress condition to that in normal BG11 medium, respectively, and a fold change > 2.0 was used as a cutoff. As shown in Table 3, expression level of most genes did not show significant changes in WT under 4.6-µM CdSO_4_ stress compared with WT cultured in normal BG11 medium, probably because that these genes did not respond to Cd^2+^ under this concentration. However, transcriptional levels of most genes were increased significantly in ALE-9.0 when CdSO_4_ was added. Particularly, the most significant changes were found in *slr0721* and *slr0798*. Although the relative expression level of *slr0721* had no significant change in ALE-9.0 under 4.6-µM CdSO_4_, it was up-regulated almost ninefold under 9.0-µM CdSO_4_. Meanwhile, *slr0798* was up-regulated 17.5-fold under 4.6-µM CdSO_4_ condition and 36.5-fold in ALE-9.0 under 9.0-µM CdSO_4_, indicating its important roles in the tolerance to Cd^2+^. Another gene, *slr1753,* was also up-regulated 2.8-fold under 4.6-µM CdSO_4,_ while 4.0-fold under 9.0-µM CdSO_4_. Transcriptional level of *ssr1480* was increased by about 2.3-fold and *sll1586* increased by about 3.5-fold in ALE-9.0 under both 4.6- and 9.0-µM CdSO_4_ stress conditions. Meanwhile, *slr0623* was found up-regulated 2.3-fold under 4.6-µM CdSO_4_ and 1.8-fold under 9.0-µM CdSO_4_. On the other hand, the expression of s*lr0454*, *slr0774,* and *slr1302* was only slightly changed in ALE-9.0 under both Cd^2+^ conditions (fold changes less than 2.0).

**Table 3:** Relative expression of mutated genes in WT and ALE-9.0 under Cd^2+^ stress

Gene	WT + 4.6-µM Cd^2+^	ALE-9.0 + 4.6-µM Cd^2+^	ALE-9.0 + 9.0-µM Cd^2+^
*slr0454*	1.213 ± 0.188	1.266 ± 0.175	1.388 ± 0.073
*slr0623*	0.723 ± 0.103	2.362 ± 0.111	1.842 ± 0.017
*slr0721*	1.154 ± 0.137	1.410 ± 0.102	8.981 ± 0.0753
*slr0774*	1.126 ± 0.077	1.483 ± 0.121	1.960 ± 0.007
*slr0798*	0.723 ± 0.084	17.503 ± 0.097	36.548 ± 0.492
*slr1302*	1.050 ± 0.067	1.737 ± 0.070	1.359 ± 0.151
*ssr1480*	1.090 ± 0.064	2.208 ± 0.136	2.385 ± 0.062
*sll1586*	1.045 ± 0.256	3.623 ± 0.181	3.362 ± 0.041
*slr1753*	0.766 ± 0.128	2.792 ± 0.008	4.078 ± 0.218

According to the above results, the expression levels of six genes, *slr0721*, *slr0798*, *slr1753, sr1480, sll1586,* and *slr0623*, were significantly induced upon the exposure to Cd^2+^ after this ALE process, indicating their relevant roles in Cd^2+^ tolerance of ALE-9.0.

### Screening of the mutated genes related to Cd^2+^ stress via knockout analysis

Gene knockout analysis was then used to evaluate the relationship of the nine mutated genes revealed by genome re-sequencing with Cd^2+^ tolerance. Relevant genes were replaced with chloramphenicol-resistance cassettes in WT and ALE-9.0 by homologous recombination, respectively (the related names of mutants were shown as ∆*X*^WT^ and ∆*X*^ALE-9.0^), and knock of neutral site *slr0168* in WT (WT-C) and ALE-9.0 (ALE-9.0-C) was selected as controls (Table 1). 4.0- or 8.0-µM CdSO_4_ was added as additional stress for knockout strains derived from WT and ALE-9.0 to eliminate the intolerance to Cd^2+^ caused by poor growth.

**Fig. 3:**
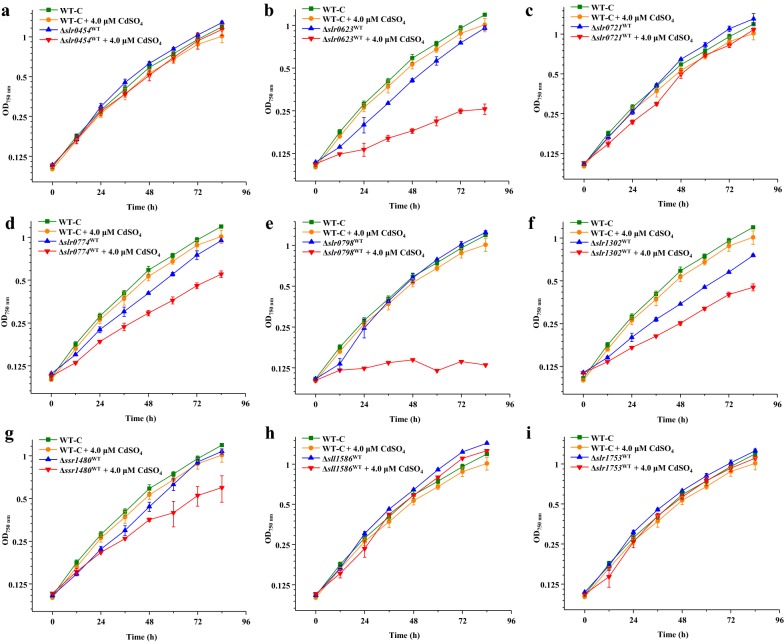
Growth patterns of WT-C and relevant knockout mutants in normal BG11 medium and under 4.0-µM CdSO_4_ at 30 °C. **a** ∆*slr0454*^WT^, **b** ∆*slr0623*^WT^, **c** ∆*slr0721*^WT^, **d** ∆*slr0774*^WT^, **e** ∆*slr0798*^WT^, **f** ∆*slr1302*^WT^, **g** ∆*ssr1480*^WT^, **h** ∆*sll158*6^WT^, **i** ∆*slr1753*^WT^. The error bars represented the calculated standard deviation of the measurements of three biological replicates

As shown in Fig. 3, ∆*slr1302*^WT^ showed significantly poor growth than WT-C in normal BG11 medium (Fig. 3d), while the others showed similar growth as WT-C, suggesting that only the knockout of *slr1302* affected the growth of WT under normal BG11 condition. Under this circumstance, it cannot be told whether *slr1302* was involved in Cd^2+^ tolerance or not. For the remained eight genes, under 4.0-µM CdSO_4_ condition, *∆slr0454*^WT^, *∆slr0721*^WT^, *∆sll1586*^WT^, and *∆slr1753*^WT^ did not show significant difference from WT-C (Fig. 3a, c, h, i), while *∆ssr1480*^WT^, *∆slr0623*^WT^, *∆slr0774*^WT^, and *∆slr0798*^WT^ demonstrated to be more sensitive to Cd^2+^ compared with WT-C (Fig. 3b, d, e, g), indicating that these four genes might be involved in Cd^2+^ tolerance in the WT strain. Among them, *∆slr0798*^WT^ could hardly grow under 4.0-µM CdSO_4_ (Fig. 3e), suggesting clearly the importance of *slr0798* to Cd^2+^ tolerance in WT.

**Fig. 4:**
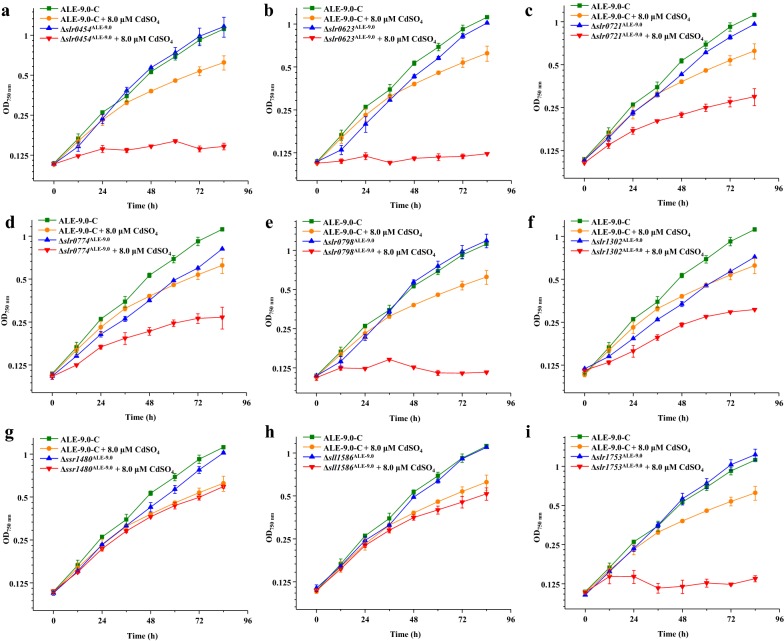
Growth patterns of ALE-9.0-C and relevant knockout mutants in normal BG11 medium and under 8.0-µM CdSO_4_ at 30 °C. **a** ∆*slr0454*^ALE-9.0^, **b** ∆*slr062*3^ALE-9.0^, **c** ∆*slr0721*^ALE-9.0^, **d** ∆*slr0774*^ALE-9.0^, **e** ∆*slr0798*^ALE-9.0^, **f** ∆*slr1302*^ALE-9.0^, **g** ∆*ssr1480*^ALE-9.0^, **h** ∆*sll1586*^ALE-9.0^, **i** ∆*slr1753*^ALE-9.0^. The error bars represented the calculated standard deviation of the measurements of three biological replicates

The nine mutated genes were also knockout individually in ALE-9.0. As shown in Fig. 4, ∆*slr1302*^ALE-9.0^ showed the same growth pattern with ∆*slr1302*^WT^ (Figs. 3f, 4f), suggesting that it also affected the growth of ALE-9.0; thus, no conclusion can be made whether *slr1302* was involved in Cd^2+^ tolerance or not. For the remained eight knockout strains, under 8.0-µM CdSO_4_ condition, *∆ssr1480*^ALE-9.0^ and *∆sll1586*^ALE-9.0^ did not show significant difference compared with ALE-9.0-C (Fig. 4g, h). Notably, the other six mutants, i.e., *∆slr0454*^ALE-9.0^, *∆slr0623*^ALE-9.0^, *∆slr0721*^ALE-9.0^, *∆slr0774*^ALE-9.0^, *∆slr0798*^ALE-9.0,^ and *∆slr1753*^ALE-9.0^ were demonstrated to be more sensitive to Cd^2+^ stress than ALE-9.0-C (Fig. 4a–e, i), indicating their vital roles in Cd^2+^ tolerance in the evolved strain ALE-9.0.

Considering the combined results of knockout analysis in both WT and ALE-9.0, *slr1302* was involved in the growth of both relative strains under normal BG11 condition though the relative mutants were more sensitive than WT or ALE-9.0 under Cd^2+^ stress condition (Figs. 3f, 4f). Therefore, it remained to be investigated whether it was also involved in Cd^2+^ tolerance or not, because the growth deficiency would also result in the sensitive phenotype under Cd^2+^ stress condition. For the remained eight genes, their involvement in Cd^2+^ tolerance did not fully match each other in WT and ALE-9.0, probably due to the different concentrations of Cd^2+^ stress, indicating the complexity of Cd^2+^ tolerance regulation mechanism in *Synechocystis*.

### Confirmation of the roles of the mutated genes in Cd^2+^ tolerance by gene overexpression in the WT strain

To evaluate the roles of these genes, especially their mutations in modifying the tolerance of Cd^2+^, the original gene in WT and their mutated genes after evolution were, respectively, overexpressed in the WT strain using an integrative vector pCP3031 with a strong promoter *Pcpc*_*560*_ [26]. The constructed strains were named OE-*X*^WT^ and OE-*X*^ALE-9.0^. Meanwhile, the empty vector of pCP3031 was overexpressed in WT and the resulted strain OE-C was set as control (Table 1). The growth patterns of these constructed strains were monitored under corresponding Cd^2+^ stress condition.

**Fig. 5:**
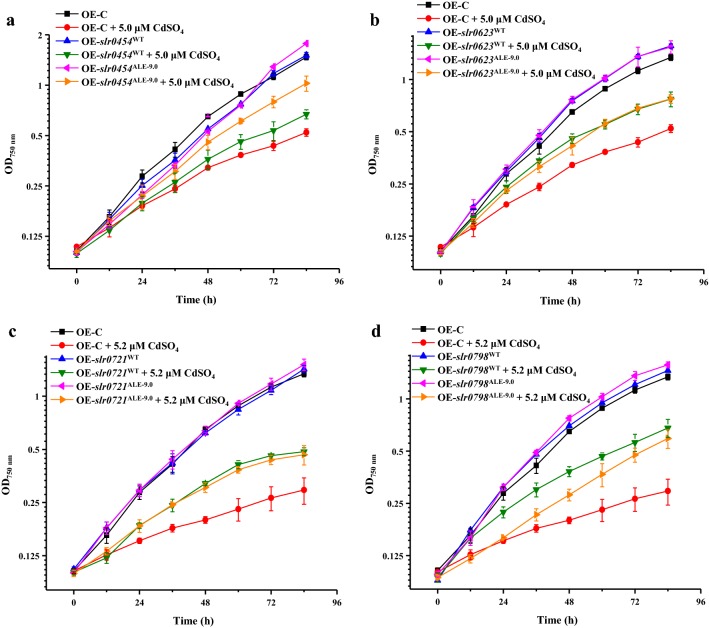
Growth patterns of OE-C and overexpression of positive genes in normal BG11 medium and under corresponding CdSO_4_ concentration at 30 °C. **a** *slr0454*, **b** *slr0623*, **c** *slr0721*, **d** *slr0798*. The error bars represented the calculated standard deviation of the measurements of three biological replicates

Among these nine genes, overexpression of four genes, *slr0454*^WT^, *slr0623*^WT^, *slr0721*^WT^ and *slr0798*^WT^, as well as their corresponding mutated genes, *slr0454*^ALE-9.0^, *slr0623*^ALE-9.0^, *slr0721*^ALE-9.0^, and *slr0798*^ALE-9.0^, successfully improved the Cd^2+^ tolerance compared with OE-C, while all these engineered strains demonstrated similar growth as OE-C in normal BG11 medium (Fig. 5a–d), further demonstrating that their expression levels were important for the Cd^2+^ tolerance. On the other hand, the overexpression of one mutated gene, *slr0454*^ALE-9.0^ exhibited better growth compared with the expression of *slr0454*^WT^, suggesting that the truncated Slr0454^ALE-9.0^ was probably more effective than Slr0454^WT^. Therefore, the results showed that both the expression level of *slr0454* and the activity of protein Slr0454 were important for Cd^2+^ tolerance, indicating the vital role of this gene in Cd^2+^ tolerance. According to the NCBI annotation, *slr0454* encodes a cation or drug efflux system protein belonging to Acriflavin–cation resistance (Acr) family. Consistent with our results, this kind of protein was reported previously to help Gram-negative bacteria to keep the intracellular homeostasis under high metal concentrations [27]. In addition, the result of protein BLAST showed that Slr0454 was homologous to inner membrane transporter, AcrB of *Escherichia coli* (*E. coli*), which has been found mediated resistance to ions including nickel, cadmium, and cobalt [28].

For the remained five genes, none of them showed positive effect on Cd^2+^ tolerance under Cd^2+^ stress condition when the WT or mutated genes were overexpressed (Additional file 1: Fig. S1a–e). Overexpression of three genes (i.e., *slr0774*^WT^, *slr0774*^ALE-9.0^, *ssr1480*^WT^, *ssr1480*^ALE-9.0^, *slr1753*^WT^, and *slr1753*^ALE-9.0^) even had negative effects on the tolerance to Cd^2+^, probably due to that the expression of these genes was already saturated, or their overexpression has brought extra metabolic burden to WT or ALE-9.0.

### Cross tolerance to other stresses obtained in ALE-9.0

**Fig. 6:**
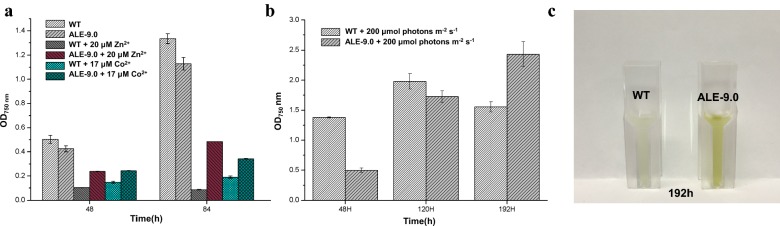
Cross tolerance of WT and ALE-9.0 against other stresses. **a** Cells growth at 48 and 84 h in normal BG11 medium, 20-µM ZnSO_4_ or 17-µM CoCl_2_. **b** Cells growth at 48, 120. and 192 h under 200-μmol photons m^−2^ s^−1^ in normal BG11 medium. **c** Colors of WT and ALE at 192 h under 200-μmol photons m^−2^ s^−1^ in normal BG11 medium

Possible cross tolerance of ALE-9.0 to other stresses was also investigated, including ZnSO_4_, CoCl_2_, CuSO_4_, high light, ethanol, and H_2_O_2_. The results showed that compared to WT, ALE-9.0 grew better under stress conditions of ZnSO_4_ and CoCl_2_ (Fig. 6a), while grew worse in other metal stresses like CuSO_4_ (Additional file 1: Fig. S2). Notably, ALE-9.0 showed better acclimation to higher illumination intensity at 200-μmol photons m^−2^ s^−1^ than WT (Fig. 6b). Although ALE-9.0 grew slower than WT at the very beginning, it was able to catch up with WT at OD_750 nm_ at day 5 and keep growing for almost 8 days, with the final OD_750 nm_ greater than WT. In addition, WT showed a bleaching phenotype, while ALE-9.0 was still yellow–green after cultured for 8 days (Fig. 6c). H_2_O_2_ and biofuel ethanol stress were also investigated, while no enhanced tolerance (actually decreased tolerance) were observed between WT and ALE-9.0 (Additional file 1: Fig. S2). Together, the results showed that along with the improved Cd^2+^ tolerance in ALE-9.0 during the ALE process, cross tolerance to Zn^2+^, Co^2+^, and high light (at late growth phase) were also obtained.

## Discussion

ALE has been demonstrated as an effective approach to obtain desired biological properties of the evolved strain. The titer of d-lactic acid produced by the evolved strain has been increased 2.0-fold than the original strain in *Leuconostoc mesenteroides* [29]. In addition, adaptive evolution under thermal stress not only increased the survival temperature from 33 to 41.5 °C, but also conferred cross tolerance to isobutanol in *Corynebacterium glutamicum* [30]. In this study, ALE was also applied to enhance Cd^2+^ tolerance of *Synechocystis* and an evolved strain tolerant to 9.0-µM CdSO_4_ was isolated after 802 day series passages. The slightly slower growth of ALE-9.0 compared with WT under normal BG11 condition (Fig. 2a, b) could be ascribed to the trade-off character to balance tolerance of higher additional stress [31]. Considering changes in color and full absorption spectrum (Fig. 2c, d), since signal near 625 nm of full absorption spectrum was measured as phycocyanin content [32] and degradation of the phycocyanin caused a color change of cyanobacterial cultures from blue–green to yellow–green [33], ALE-9.0 demonstrated less phycocyanin than WT. In addition, phycocyanin was the major part of phycobilisomes for harvesting light and causing energy migration toward photosystem reaction centers [34]. Meanwhile, early study showed that lack of phycocyanin would result in poor light-harvesting ability [24]. Thus, we speculated that the light-harvesting ability of ALE-9.0 became weaker after long-time exposure to Cd^2+^.

ALE always brings about global changes at whole-genomic, transcriptional, and metabolomic levels [35]. In a previous research with *Synechocystis*, an acid-evolved strain identified 11 mutations in the genome, and the transcriptional differences were demonstrated by qRT-PCR [36]. A recent study improved isobutanol tolerance from 2 to 5 g/L with combinatorial malfunctions of three genes [37]. Consistently, in this study, nine mutations were identified by the whole-genome re-sequencing. Combined with knockout and overexpression analysis, out of these nine mutations, only the SV in *slr0454* was demonstrated to improve the tolerance directly (Fig. 5a). As mentioned above, Slr0454 was homologous to AcrB. It was reported that AcrB cooperated with a membrane fusion protein AcrA and an outer membrane channel TolC to form an AcrAB–TolC system to export drugs [38]. In this study, the results also showed that the truncated Slr0454 contributed to tolerance of Cd^2+^ in *Synechocystis*, consistent with a previous study in *Synechococcus* sp. PCC 7942 showing the truncated form of the bacterial heat shock protein ClpB contributed to development of thermotolerance [39], probably by transforming the protein structure into a more effective conformation.

On the other hand, five mutated genes, *slr0623*, *slr0721*, *slr0798*, *slr0774,* and *slr1753,* were demonstrated to be involved in the Cd^2+^ tolerance in ALE-9.0 via knockout analysis (Fig. 4b–e, i). Among these five genes, although no positive effects were shown in the overexpression analysis between the WT genes and mutated ones, the increased expression of *slr0623*, *slr0721,* and *slr0798* was demonstrated to contribute to the increased Cd^2+^ tolerance in ALE-9.0 (Table 3, Fig. 5b–d). *slr0623* encodes thioredoxin (Trx) TrxA, which is a class of small redox proteins known to be present in most microorganisms. Consistent with our results, research in *E. coli* showed Trx was inhibited by Cd^2+^ and posed a positive role in protection from Cd^2+^ [40]. According to this study, Cd^2+^ directly bound to Trx by forming a chelator and decreased thiol-disulfide transferase activity, and this kind of Cd^2+^ sink might help against Cd^2+^ stress. *slr0721* showed similar results as *slr0623* both in gene knockout and overexpression analysis. *slr0721* encodes the decarboxylating NADP-dependent malic enzyme, participating in tricarboxylic acid cycle. A study on malic acid and Cd^2+^ stress in *Miscanthus sacchariflorus* proved exogenous addition of malic acid could alleviate Cd^2+^ toxicity through enhancing photosynthetic capacity and restraining ROS accumulation [41]. Even though this phenomenon was widely discovered in plants [42, 43], we supposed that similar tolerance mechanism might also be utilized in cyanobacteria, while further evidences are still needed. It was noteworthy that expression level of *slr0721*^*ALE*-*9.0*^ in ALE-9.0 did not show significant change under 4.6 µM, but was increased significantly under 9.0-µM CdSO_4_ (Table 3). Meanwhile, Sanger sequencing showed that the mutation of *slr0721* occurred in later stage after 7.0 µM (Table 2), indicating the possibility of different response mechanism that this gene was involved to Cd^2+^ stress. Finally, Slr0798 is an SmtB-like repressor concerning zinc-transporting P-type ATPase involved in zinc tolerance [44]. Consistent with the previous study in *Synechocystis*, overexpression of *slr0798* gene with a replicative vector pJA2 could significantly enhance Cd^2+^ tolerance [23]. Unlike SmtB, Slr0798 triggered excess Zn^2+^ expulsion by via Slr0798-mediated efflux into the periplasm, which we supposed was the same mode for Cd^2+^. According to the qRT-PCR analysis (Table 2), the expression of *slr0798*^*ALE*-*9.0*^ was significantly up-regulated under CdSO_4_ stress, suggesting the importance of expression level of this gene in Cd^2+^ tolerance. The other two mutated genes, *slr0774* and *slr1753* shared similar functions related to membrane protein. These two genes showed involvement in Cd^2+^ tolerance in the knockout analysis, but neither could improve tolerance directly according to the overexpression analysis, probably due to that the expression of these genes was already saturated for Cd^2+^ tolerance. *slr0774* encodes SecD, a part of Sec protein. The general secretory (Sec) pathway was considered as a major translocation process of protein from cytosol across the cytoplasmic membrane in bacteria [45] and SecD acted as an auxiliary component to enhance translocation efficiency [46]. It is then speculative that the mutation of *slr0774* could lead to different efficiency of SecD or help SecD interact with other membrane protein better, leading to high Cd^2+^ tolerance. In addition, *slr1753* was found as an outer membrane fraction for its homology to a cell-surface glycoprotein in *Clostridium thermocellum* [47]. Besides glycoprotein interacted selectively and non-covalently with carbohydrate and increase of EPS production enhanced Cd^2+^ resistance [48], it is thus supposed that the mutation of *slr1753* possibly was able to help optimize the constitution and content of saccharide on cell-surface, leading to different Cd^2+^ tolerances. Since the ALE-9.0 could tolerate 9.0-µM Cd^2+^, except for the function of each single gene, the combinations of these genes and mutations to work together most probably also exist [30] which is still yet to be determined.

Mutations occur randomly during the course of ALE and are selected naturally when a particular mutation enhances the activity of a protein and/or thereby the better tolerant or survival [37]. For Cd^2+^ tolerance, some genes including *slr0649*, *slr0946*, *slr1738,* and *sll1598* were also demonstrated to be related to Cd^2+^ tolerance in the previous studies [8]. However, these genes were not found to mutate during our ALE process, which might be due to, we supposed, the randomness of ALE experiment, and meanwhile, on the other hand, it was also possible that although these genes did not mutate, their expression level might change, which remains to be investigated.

As for cross tolerance, the evolved ALE-9.0 also obtained cross tolerance to Zn^2+^, Co^2+^, and high illumination intensity (Fig. 6a, b). Zn^2+^ belongs to the same group as Cd^2+^, which have the same chemical properties, consistent with that some mutated genes in ALE-9.0 like *slr0798* also showed involvement in Zn^2+^ tolerance [8]. In addition, the previous studies have found that Cd^2+^ and Co^2+^ shared some similar toxicity mechanism [49] and Acr family showed resistance to Co^2+^ too [28], consistent with the finding that *slr0454* involved in Co^2+^ tolerance. In addition, this was also confirmed in our experiments by the result that a higher tolerance to Co^2+^ was exhibited in ALE-9.0 (Fig. 6a). For high light, ALE-9.0 was hypothesized less sensitive to strong light than WT because of the poor light-harvesting ability [22]. At first, WT grew quickly as a result of enough light and ample carbon resource for downstream reaction, but later, the accumulation of ROS emerged became dominant [50] and caused the photo damage [24], while ALE-9.0 could absorb enough light only for growth and strong light did less harm to it, which also resulted in larger biomass of ALE-9.0 than WT (Fig. 6b). In addition, our results showed that ALE-9.0 did not demonstrate enhanced tolerance (actually even decreased tolerance, Additional file 1: Fig. S2) to H_2_O_2_. Although Cd^2+^ induces stresses including oxidative stress, tolerance of strains to Cd^2+^ involves many aspects like ion efflux and chelation [51], so it can be supposed that during this ALE process, the enhanced Cd^2+^ tolerance of ALE-9.0 may not involve oxidative tolerance. Besides, the ALE-9.0 grew slower than WT under normal condition, which may also result in the sensitiveness to most of other unrelated stresses such as H_2_O_2_.

## Conclusion

In this study, an evolved strain ALE-9.0 of *Synechocystis* that could tolerate up to 9-µM CdSO_4_ after 802 day ALE process was obtained. The mutations in the genome of ALE-9.0 compared with WT were identified by genome re-sequencing. One mutation of *slr0454* was demonstrated capable of improving Cd^2+^ tolerance directly and five mutated genes, *slr0623*, *slr0721*, *slr0798*, *slr0774,* and *slr1753,* were demonstrated involved in the Cd^2+^ tolerance in ALE-9.0. In addition, the evolved ALE-9.0 also obtained cross-tolerance ability to Zn^2+^, Co^2+^, and high light. Our work here identified six genes related to Cd^2+^ tolerance and demonstrated the feasibility of ALE in tolerance modifications. This work also provided valuable information to decipher the cadmium tolerance mechanism in *Synechocystis* and useful insights for cyanobacterial robustness and tolerance engineering.

## Methods

### Bacterial growth conditions

The wild-type *Synechocystis*, laboratory-evolved, and constructed strains were grown on BG11 agar plate or in BG11 medium (pH 7.5) under a light intensity of approximately 50-μmol photons/m^2^/s in an illuminating or shaking incubator of 130 rpm at 30 °C (HPX-9162 MBE, BOXUN, China, HNY-211B Illuminating Shaker, Honour, China) [52]. Proper antibiotic was added to maintain the stability (i.e., 20-μg/mL spectinomycin, 20-μg/mL chloramphenicol) of the constructed strains. Cell optical density and full absorption spectrum were monitored by a UV-1750 spectrophotometer (Shimadzu, Japan) at 750 nm. *E. coli* strain DH 5α was used for constructing and collecting plasmids. *E. coli* was grown on LB agar plate or in LB liquid medium in incubator at 37 °C or shaking incubator at 200 rpm supplemented with appropriate antibiotic (i.e., 50-μg/mL spectinomycin, 50-μg/mL chloramphenicol, and 200-μg/mL ampicillin).

### Adaptive laboratory evolution of Cd^2+^ tolerance

Adaptive laboratory evolution of Cd^2+^ tolerance was carried out in 20 mL liquid BG11 medium in a 100 mL shake flask. The CdSO_4_ stock solution was prepared with CdSO_4_·8/3H_2_O of analytical pure, purchased from Aladdin (Shanghai, China). The initial WT stain was cultured with 4.6-µM CdSO_4_ from an inoculum of OD_750 nm_ 0.1. Cd^2+^ concentration in BG11 medium was increased by 0.3–0.4 µM when the culture reached OD_750 nm_ of 0.5 within 96 h. The simplified process is shown in Fig. 1b. To exclude the potential effects of the residual Cd^2+^ in the last passage, the culture was centrifuged and transformed into fresh BG11 medium during evolution. Serial adaptation passages were conducted until the final tolerance to CdSO_4_ achieved 9.0 µM. After confirming that the strain can survive under 9.0-µM CdSO_4_, the evolved strain was screened on BG11 agar plate with 9.0-µM CdSO_4_, and four clones were isolated and re-cultured in BG11 liquid medium. After re-confirmation of the tolerance, one clone showing greatest growth state was selected for further analysis (Fig. 1a).

### Whole-genome re-sequencing

Isolation of genomic DNA was carried out as described previously [53]. Total DNA obtained was subjected to quality control by agarose gel electrophoresis and quantified by Qubit. The genome of WT strain and the evolved strain ALE-9.0 were sequenced with MPS (massively parallel sequencing) Illumina technology by a paired-end library with an insert size of 350 bp. This 350-bp library was sequenced using an Illumina HiSeq4000 by PE150 strategy. Original figure data obtained by high-throughput sequencing were transformed into raw sequenced reads (raw data, or raw reads). Then, sequenced data were filtered and the sequence of adapter and low-quality data were removed, resulting in the clean data used for subsequent analysis. Variation information of the sample and the reference is obtained by aligning the sample reads with the designated reference (https://www.ncbi.nlm.nih.gov/nuccore/NC_000911). Final results involved SNP (single-nucleotide polymorphism), InDel (insertion and deletion of small fragments in the genome), and SV (insertion, deletion, inversion, and translocation of the large segments in the genome level).

### Sanger sequencing

To validate the SNP, InDel, and SV revealed by re-sequencing, Sanger sequencing was performed. Primers used to amplify gene fragments are listed as “primers for Sanger sequencing” in Additional file 2: Table S1. The gene fragments were then ligated to EZ-T™ (GENSTAR, Beijing, China) by original TA cloning kit and the plasmids obtained were sent for sequencing.

### Strains’ construction

Strains and plasmids used in this study are listed in Table 1. Among them, *E. coli* DH5α was used for vector construction and amplification. For knockout of relative original and mutated genes, the plasmids’ framework was obtained from above plasmid used for sequencing by PCR with primers for gene knockout (Additional file 2: Table S1), and then ligated with chloramphenicol-resistance cassette (amplified from a plasmid pACYC184). Then, the constructed plasmid was transformed into *Synechocystis* by natural transformation [54].

For genes overexpression, an integrative vector pCP3031 with spectinomycin-resistant cassette was used [26]. Relative genes were first obtained by PCR. Primers for gene overexpression are listed in Additional file 2: Table S1. Afterwards, target genes were ligated into pCP3031. The constructed plasmid was finally transformed into *Synechocystis* by natural transformation [54].

Both knockout and overexpression strains were verified by PCR and sequencing analysis.

### qRT-PCR analysis

The qRT-PCR analysis was used to compare the gene expression level between strains grown in normal and Cd^2+^ stress conditions. Primers for qRT-PCR analysis were designed by Primer Express 2.0 and listed in Additional file 2: Table S1. Experimental steps were based on the description as previously [55]. Three technical replicates were used for each sample. Data analysis was performed via the StepOnePlus analytical software (Applied Biosystems, Foster City, CA, United States) and the 2^−*∆∆*CT^ method [56]. The 16s RNA was used as an internal reference. Data were shown as ratio of the amount of genes’ transcript in WT or ALE-9.0 under Cd^2+^ stress to those cultured in normal condition without Cd^2+^ stress, respectively.

### Growth profile analysis

To monitor growth profile under Cd^2+^, fresh cells were collected by centrifugation and then inoculated into 20 mL of BG11 liquid medium in a 100-mL flask. Three biological parallels were used for each sample. The initial concentration of cells was adjusted at OD_750 nm_ of 0.1. Then, culture samples were taken and measured at OD_750 nm_ every 12 h. For knockout mutants in WT, 4.0-µM CdSO_4_ was added, while for knockout mutants in ALE-9.0, 8.0-µM CdSO_4_ was added. For overexpression strains, three different concentration levels (i.e., 4.8, 5.0, and 5.2 µM) were set out for different genes.

Growth under other examined stress conditions was also measured in the same way as above. Concentrations of chemicals used were as follows: 20 µM for ZnSO_4_, 1.8 µM for CuSO_4_, 17 µM for CoCl_2_, 1 mM for H_2_O_2,_ and 1 mM for ethanol. High light was set as 200-μmol photons/m^2^/s.

## Additional files

**Additional file 1: Fig. S1.** Growth patterns of OE-C and overexpression of other genes in normal BG11 medium and under corresponding CdSO_4_ concentration. (a) *slr0774*, (b) *slr1302*, (c) *ssr1480*, (d) *sll1586*, (e) *slr1753*. The error bars represented the calculated standard deviation of the measurements of three biological replicates. **Fig. S2.** Cross tolerance of WT and ALE-9.0 against other stresses. Cell growth at 48 and 84 h in normal BG11 media, 2% ethanol, 1.8 μM CuSO_4_ or 1 mM H_2_O_2_. ALE: adaptive laboratory evolution.

**Additional file 2: Table S1.** All the primers used in this study.

## Abbreviations

Acr: acriflavin–cation resistance
ALE: adaptive laboratory evolution
Cd^2+^: cadmium ion
ROS: reactive oxidative species
Trx: thioredoxin
WT: wild type
