# Supplementary material for: Adaptive laboratory evolution of cadmium tolerance in Synechocystis sp. PCC 6803
Source: Biotechnol Biofuels. 2018 Jul 24;11:205. doi: 10.1186/s13068-018-1205-x (PMC6058365; doi:10.1186/s13068-018-1205-x)
Supplement: Supplementary file 1 — Additional file 1: Fig. S1. Growth patterns of OE-C and overexpression of other genes in normal BG11 medium and under corresponding CdSO4 concentration. (a) slr0774, (b) slr1302, (c) ssr1480, (d) sll1586, (e) slr1753. The error bars represented the calculated standard deviation of the measurements of three biological replicates. Fig. S2. Cross tolerance of WT and ALE-9.0 against other stresses. Cell growth at 48 and 84 h in normal BG11 media, 2% ethanol, 1.8 μM CuSO4 or 1 mM H2O2. ALE: adaptive laboratory evolution. [file 13068_2018_1205_MOESM1_ESM.pptx]

## Slide 1
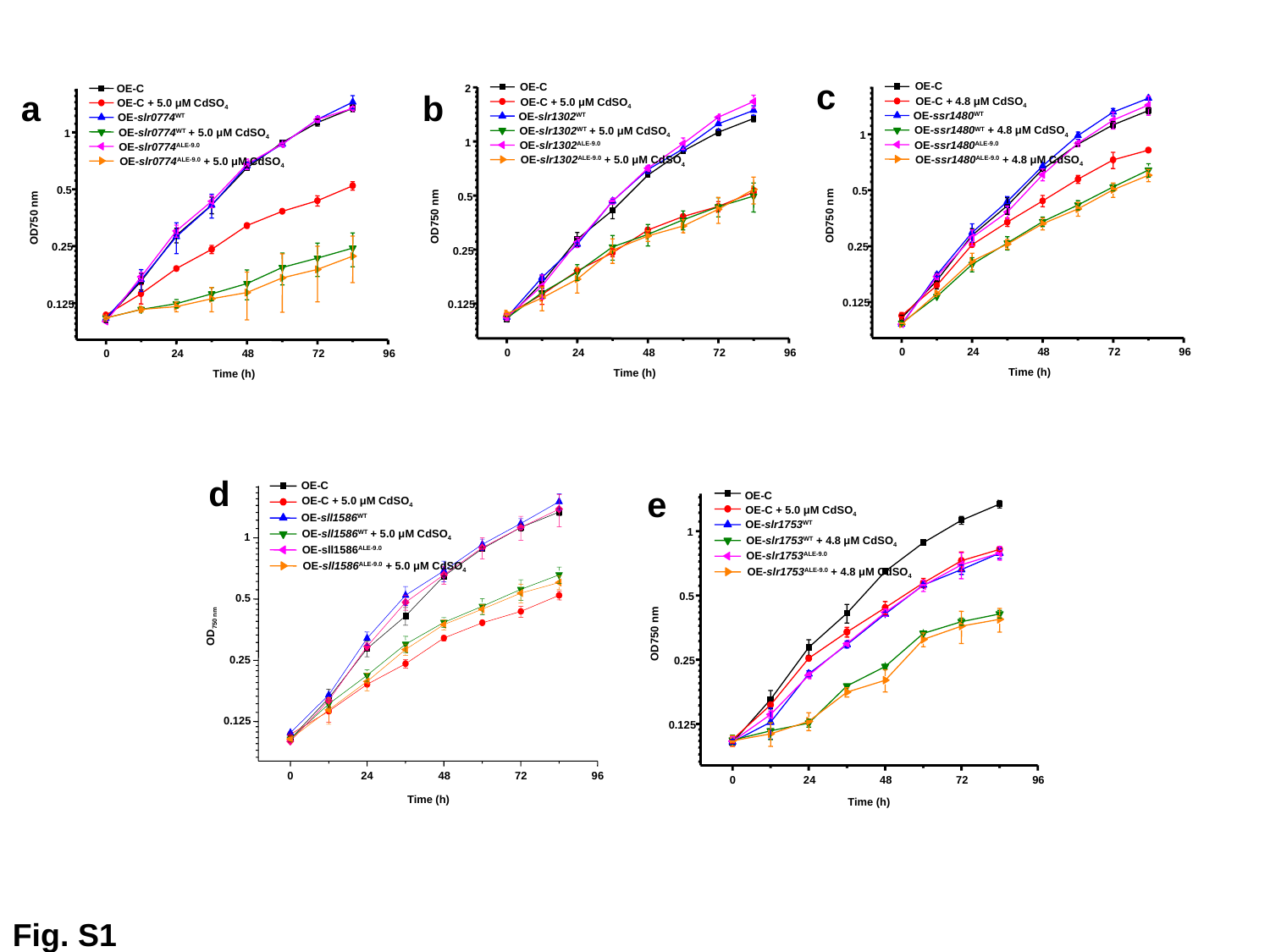

1
0.5
0.25
0.125
0
24
48
72
96
OE-ssr1480WT
OE-ssr1480WT + 4.8 μM CdSO4
OE-ssr1480ALE-9.0
OE-ssr1480ALE-9.0 + 4.8 μM CdSO4
OD750 nm
Time (h)
2
1
0.5
0.25
0.125
0
24
48
72
96
OE-slr1302WT
OE-slr1302WT + 5.0 μM CdSO4
OE-slr1302ALE-9.0
OE-slr1302ALE-9.0 + 5.0 μM CdSO4
OD750 nm
Time (h)
OE-C
1
0.5
0.25
0.125
0
24
48
72
96
OE-C + 5.0 μM CdSO4
OE-slr0774WT
OE-slr0774WT + 5.0 μM CdSO4
OE-slr0774ALE-9.0
OE-slr0774ALE-9.0 + 5.0 μM CdSO4
OD750 nm
Time (h)
c
OE-C
a
OE-C
b
OE-C + 4.8 μM CdSO4
OE-C + 5.0 μM CdSO4
1
0.5
0.25
0.125
0
24
48
72
96
OE-slr1753WT
OE-slr1753WT + 4.8 μM CdSO4
OE-slr1753ALE-9.0
OE-slr1753ALE-9.0 + 4.8 μM CdSO4
OD750 nm
Time (h)
d
e
1
0.5
0.25
0.125
0
24
48
72
96
OD750 nm
OE-sll1586WT
OE-sll1586WT + 5.0 μM CdSO4
OE-sll1586ALE-9.0
OE-sll1586ALE-9.0 + 5.0 μM CdSO4
Time (h)
OE-C
OE-C
OE-C + 5.0 μM CdSO4
OE-C + 5.0 μM CdSO4
Fig. S1

## Slide 2
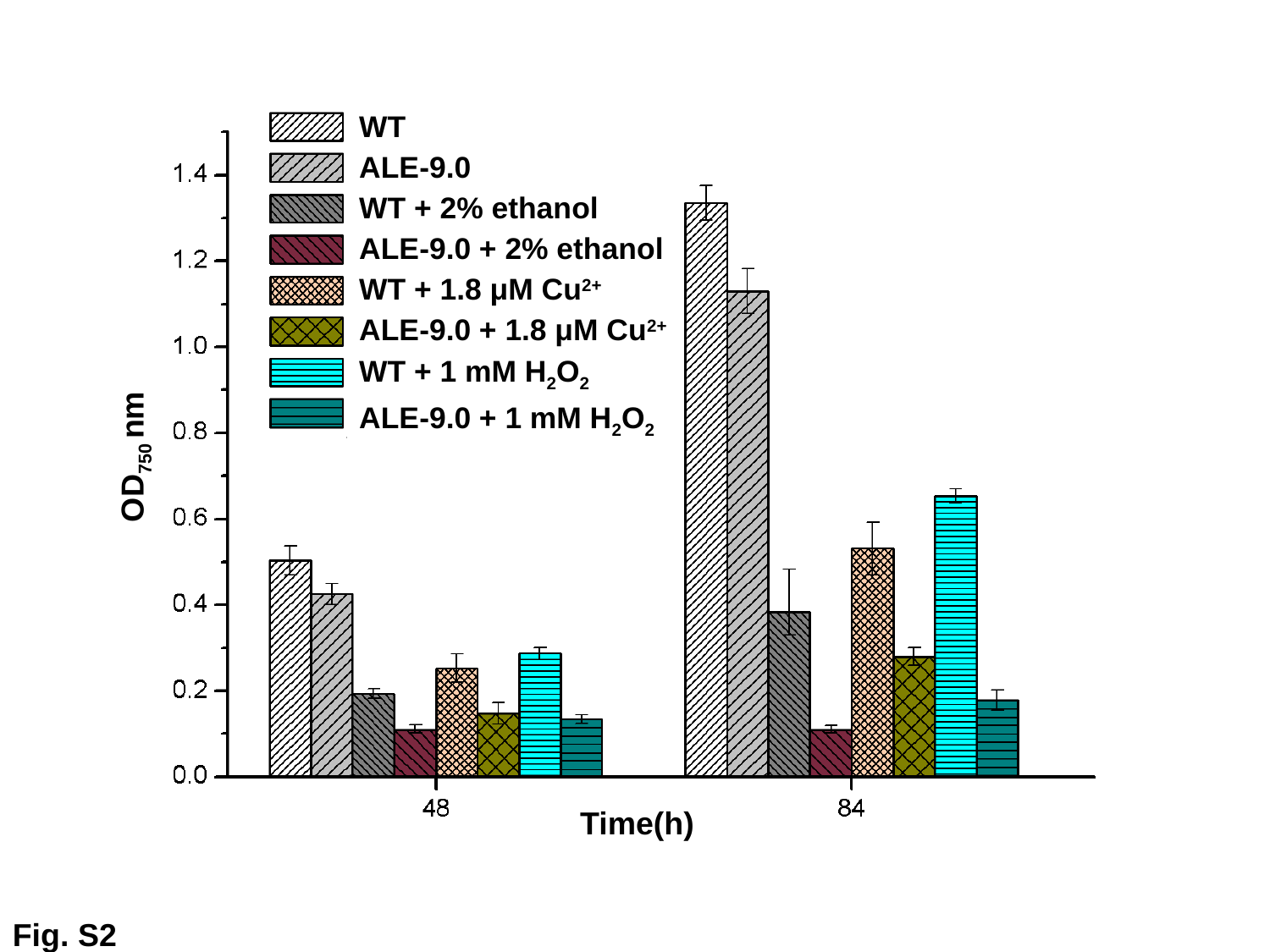

WT
ALE-9.0
WT + 2% ethanol
ALE-9.0 + 2% ethanol
WT + 1.8 μM Cu2+
ALE-9.0 + 1.8 μM Cu2+
WT + 1 mM H2O2
ALE-9.0 + 1 mM H2O2
OD750 nm
Time(h)
Fig. S2
