# Supplementary material for: Adaptive laboratory evolution of cadmium tolerance in Synechocystis sp. PCC 6803
Source: Biotechnol Biofuels. 2018 Jul 24;11:205. doi: 10.1186/s13068-018-1205-x (PMC6058365; doi:10.1186/s13068-018-1205-x)
Supplement: Supplementary file 2 — Additional file 2: Table S1. All the primers used in this study. [file 13068_2018_1205_MOESM2_ESM.docx]

| **Table S1. All the primers used in this study.** | |
| --- | --- |
| **Primers for Sanger sequencing** | |
| *slr0454-F* | AATCCGCTCCCCCCAAAATTG |
| *slr0454-R* | GGTGGTCATCAAAATGGGGCG |
| *slr0623-F* | AATTGCTCCCCCAGTTTTTCCAG |
| *slr0623-R* | TCTGCAATGTCCCATACCAGCG |
| *slr0721-F* | AGGTATTGATGATGGTACGGGCTG |
| *slr0721-R* | GCGGGTAATACGGGCATTGG |
| *slr0774-F* | AACCAATTACTGAGCAGGGCAATG |
| *slr0774-R* | ACGGGTCAAAGTTTGATTGACTGC |
| *slr0798-F* | ACTTGCCATCCCCAGATAATGGAG |
| *slr0798-R* | TGGTGGTTAGCAGGCTTAGCC |
| *slr1302-F* | TCTCTATCGATTGGCCACCGGC |
| *slr1302-R* | CTCCGTAGACGGGATGGTTCTG |
| *ssr1480-F* | ATCTCAAAGCCATTAACCCCAACG |
| *ssr1480-R* | AGAATGGCCGCATCTTCTACCC |
| *sll1586-F* | CGCAGGCATTGACGTCTATTCC |
| *sll1586-R* | TTTGCAGTGGCCCGGTTTTG |
| *slr1753-F* | AATGCGGATGCGCTCATTGTC |
| *slr1753-R* | GGCTAAACCCCAAAAGGTATCCGG |
| **Primers for gene knockout** | |
| *as-slr0454-F* | TTTCCTCAGTGCCAATTTTGGCG |
| *as-slr0454-R* | CGGGTTCTAGCTTGGTCTTTGG |
| *as-slr0623-F* | CTTGAGGGGTAGCACTCATACTGG |
| *as-slr0623-R* | CTTTAAGCGCTGGCTTCCCT |
| *as-slr0721-F* | AGGGCGATGGAAAAAGGGTTT |
| *as-slr0721-R* | TGGCCGGTCAATAGTTAATCGGG |
| *as-slr0774-F* | GGGAAAGGAATTGATCGGGGACA |
| *as-slr0774-R* | CGGTAACGGCTAAGTCTTAAAAGCT |
| *as-slr0798-F* | GGCGGCCAACGTGATTTAAAGA |
| *as-slr0798-R* | AGTGCATTGACAGTATCCATACCCT |
| *as-slr1302-F* | AAGGAAATTTCTTCAGCCCCAATCG |
| *as-slr1302-R* | CCGGCTCTCCATCAGCACT |
| *as-ssr1480-F* | GGCTTGGTAAGAAAGGTTCCCGA |
| *as-ssr1480-R* | GACCGAGAACCCCTCGTTAAGT |
| *as-sll1586-F* | CCTTTGCTAGCCGGAGGTCTC |
| *as-sll1586-R* | AATTTCAGGCGCTATTCATTCCCC |
| *as-slr1753-F* | AAAGCCCAGGGAAGGGACTTTTTG |
| *as-slr1753-R* | AGTGGGCAGCCCTTGGTAAC |
| **Primers for gene overexpression** | |
| *Xho-slr0454-F* | CCCTCGAGGTGCGAATCAATTCATCTTCAAAGAATCGT |
| *Nde-slr0454-R* | CCCATATGTCAGGTTTTTTCCGGCCTGGG |
| *Xho-slr0623-F* | CCCTCGAGATGAGTGCTACCCCTCAAGTTTCC |
| *Bgl-slr0623-R* | GAAGATCTTTAAAGATATTTTTCTAGGGTGCTGGCCAG |
| *Xho-slr0721-F* | CCCTCGAGATGGTTAGCCTCACCCCCAA |
| *Bgl-slr0721-R* | GAAGATCTCTATTGACCGGCCACCCC |
| *Xho-slr0774-F* | CCCTCGAGATGCAAAGACTGCGTTGGCTAC |
| *Bgl-slr0774-R* | GAAGATCTTTAAGACTTAGCCGTTACCGATGATAGATTC |
| *Xho-slr0798-F* | CCCTCGAGATGACCCAATCTTCACCGCTCA |
| *Bgl-slr0798-R* | GAAGATCTTTACTTAGCAATCCGAGTAGCATTCAAAATCG |
| *Xho-slr1302-F* | CCCTCGAGGTGCCCTGGCATTCTGGC |
| *Bgl-slr1302-R* | GAAGATCTCTAATCGGCCATGGTTGAGGG |
| *Xho-ssr1480-F* | CCCTCGAGATGTCCATTTATGTCGGGAACCTTTCTT |
| *Bgl-ssr1480-R* | GAAGATCTTTAACGAGGGGTTCTCGGTCTTG |
| *Xho-sll1586-F* | CCCTCGAGATGGTCACCGATCGCCAAAAG |
| *Bgl-sll1586-R* | GAAGATCTCTAAAACCTTTTTTGAAATTCCACTATGCCCC |
| *Xho-slr1753-F* | CCCTCGAGGTGGTGGAAATATTGTTAATTTCACCCCT |
| *Bgl-slr1753-R* | GAAGATCTTTACCAAGGGCTGCCCACTAG |
| **Primers for qRT-PCR** | |
| *qRT-slr0454-F* | CTGTTGGGAGCGATCGTCTT |
| *qRT-slr0454-R* | CCCAACCGCTACGGAGAAA |
| *qRT-slr0623-F* | AGTCAGTATGGCATCCGCAGTA |
| *qRT-slr0623-R* | TCTCTGGCCCCCCTTGA |
| *qRT-slr0721-F* | CCGGAGCCACCGATATTTG |
| *qRT-slr0721-R* | TGCGATGTTTGCCCACAA |
| *qRT-slr0774-F* | CGCAGCGGTAATTACAGGAAA |
| *qRT-slr0774-R* | TGCACCGCCAGATCGTT |
| *qRT-slr0798-F* | CGCCGTTCCATCAAAGCT |
| *qRT-slr0798-R* | TGTACCATTGCGCTTCAAGTTC |
| *qRT-slr1302-F* | TGGTGGGCATCCTCAAAAG |
| *qRT-slr1302-R* | TGTGTTGCAGATTGATGGAATAGG |
| *qRT-ssr1480-F* | GGCACTGTTAAGCGGGTTCA |
| *qRT-ssr1480-R* | AACCAAAACCCCGCATACG |
| *qRT-sll1586-F* | GATCAAGGCAACCCAACAAAG |
| *qRT-sll1586-R* | AACCTACTCAGCCGTGGACAA |
| *qRT-slr1753-F* | AGGGCGGCTCCTTTTCC |
| *qRT-slr1753-R* | TTGCTCGCTCCTTGGGTTA |
| *qRT-16S-F* | TGTAGCGGTGAAATGCGTAG |
| *qRT-16S-R* | CCACGCCTAGTATCCATCGT |
